# AI-powered smart emergency services support for 9-1-1 call handlers using textual features and SVM model for digital health optimization

**DOI:** 10.3389/fdata.2025.1594062

**Published:** 2025-07-07

**Authors:** Afraa Attiah, Manal Kalkatawi

**Affiliations:** Department of Information Technology, Faculty of Computing and Information Technology, King Abdulaziz University, Jeddah, Saudi Arabia

**Keywords:** digital healthcare, 9-1-1 call handling, machine learning, natural language processing (NLP), decision support, real-time analysis

## Abstract

In emergency situations, 9-1-1 is considered the first point of contact, and their call handlers play a crucial role in managing the emergency response. Due to the large number of daily calls and the hectic routine, there are severe chances that the call handlers can make any mistake or human error during data taking in a high-pressure environment. These mistakes or errors impact 9-1-1 performance in emergencies. To address this problem, this research introduces an AI-powered digital health framework called Emergency Calls Assistant (ECA) that leverages artificial intelligence (AI) and natural language processing (NLP) techniques to assist call handlers during data collection. ECA is designed to predict the type of emergency, suggest relevant questions to collect deeper information, suggest pre-arrival instructions to emergency personnel, and generate incident reports that helps in data-driven decision making. The ECA framework works in two phases; the first phase is to convert the audio call into digital textual form, and the second phase is to analyze the textual information using NLP tools and mining techniques to retrieve contextual information. The second phase also deals with emergency categorization using a support vector machine (SVM) learning model to prioritize the emergency dealing with an accuracy of 92.7%. The key factors involved in categorization by ML models are the severity of injury and weapons involvement. The objective of ECA's development is to provide digital health-saving technology to 9-1-1 call handlers and save lives by making accurate decisions by providing real-time assistance. This research aligns with the advancement of digital health technologies by exhibiting how NLP-driven decision support systems can revolutionize emergency healthcare, improve patient outcomes through real-time AI integration, and reduce errors.

## 1 Introduction

In emergencies, whether it is medical issues, traffic accidents, or crimes, the most important thing is to provide early and accurate services to personnel. In major cities, this emergency service is provided by the 9-1-1 call handler that plays a major role in the initial discussion and healthcare service providing. As 9-1-1 call handlers receive a huge amount of calls each day, there are severe chances of errors in their data analysis under a high-pressure environment that can impact the service and result in the death of that personnel (Skryabina et al., 2017). Due to the intense demands of this position, cutting-edge support systems are essential to assist call handlers in making quick and accurate decisions.

Emergency call-handling systems are currently robust but face inherent limitations. These systems largely depend on the manual expertise of call handlers to categorize emergencies and offer suitable instructions (Maletzki et al., 2022). However, the growing volume and complexity of emergency calls necessitate a more advanced approach to assist call handlers, minimize errors, and improve response times. The 9-1-1 number is used in many countries for dealing with emergencies by connecting multiple departments like fire-extinguisher teams, traffic police, and ambulance services. As 9-1-1 plays a central role in connecting these departments, it is also their responsibility to provide each department with information on emergencies by communication and information sharing. In Saudi Arabia, the Ministry of Interior reveals that around 2.5 million calls are received on 9-1-1 per month. It is approximately equal to 58 calls per minute. Around 15–20 of incoming 9-1-1 calls withing that one minute are non-emergency (Ministry of Interior, Saudi Arabia, 2014). This type of pressure urges the demand of an automated and AI with NLP assisted framework for taking appropriate notes and directing them to the relevant departments for an effective response.

Several automated systems have been developed to enhance the efficiency of 9-1-1 services and reduce response times. For example, RapidSOS (RapidSOS, 2012) is another emergency response platform integrates with 9-1-1 systems to provide critical data analytics during emergency calls. RapidSOS shares medical information, real-time location, and accident data with 9-1-1 call centers. This leads to helping first responders assess and respond quickly to emergencies. Corti (2016) also uses AI to assist emergency services by analyzing emergency calls in real-time. The Corti-AI listens to the calls, detects severe medical conditions like heart attacks, and provides real-time alerts to medical emergency response team. Moreover, AlertGO (2017) is another emergency response management system designed to enhance the efficiency of emergency call handling. It leverages advanced analytics and real-time data processing to prioritize and dispatch resources effectively. These systems and some others were studied thoroughly, and we found many limitations that need to be addressed in a more comprehensive system.

Receiving multiple calls daily and responding to them properly with appropriate department help is stressful. According to National Emergency Number Association (NENA), the U.S. 9-1-1 system receives an average of about 600,000 calls daily and ~240 million calls annually (National Emergency Number Association (NENA), 2024). Sometimes, more than two calls operate at the same time, and it is difficult for a 9-1-1 call handler to decide which one to address first. The department to which the query is forwarded also demands appropriate data and asks multiple questions to address the problem accordingly. Another responsibility of the call handler is to remain on call and deal with caller queries until the real help arrives. Furthermore, call handlers must be experts in different fields to get accurate information from the caller and forward it to the concerned department. Inaccurate information could cause a delay in the arrival to the caller's location. The major contributions of this research work are:

- The Objective of this research work is to build an intelligent system called Emergency Call Assistant (ECA) that will support the call handler in making life-saving decisions by using SVM ML model, NLP techniques, and front-end development for the interface.
- The ECA system operates in a real-time environment by getting caller personal information, predicting emergency type, and making accurate categorizing predictions based on emergency severity. The accuracy rate is 92.7%.
- The proposed framework automatically detects keywords from the caller voice converted to textual form for report generation to help the concerned department to act accordingly.

The paper proceeds as follows: Section 2 reviews the related work in emergency calls handling applications. Section 3 explains the dataset and discusses ECA, the proposed model. Moreover, we discuss the experiments in Section 4, while Section 5 concludes the paper.

## 2 Related work

In emergencies, the 9-1-1 call center service is of utmost importance. The 9-1-1 call center plays a pivotal role in ensuring appropriate and timely response to emergencies. This quick response centers results in saving lives and mitigating critical situations. Multiple frameworks have been developed to improve the effectiveness and efficiency of 9-1-1 services. Some studies improve the efficacy of emergency services by integrating ML techniques (Mitchell, 1997). This integration aims to assist 9-1-1 call handlers in their critical tasks by enabling them to handle emergency calls efficiently and make informed decisions. In Wang et al. (2023), authors develop an ML model to improve ambulance dispatch triage for Emergency Medical Services (EMS). This also extends the integration of ML in various other EMS fields like survival prediction, symptom recognition, and patient transportation to enhance the accuracy of medical dispatch systems. This research also emphasizes the importance of using multi-modal (video and patient health records) data to further transform EMS. This research work also has some limitations like a lack of hospital data and using retrospective single-center data, which results in a significant reduction in over-triage rates by over 5% while maintaining similar under-triage levels.

Furthermore, Shukhman and Shukhman (2022) discusses the potential of ML and NLP in enhancing the efficiency of unified duty and dispatch services. The authors explore various methods of text classification for automatic categorization of emergency messages into three classes: fire service, ambulance service, and police service. Maletzki et al. (2023) proposed approach builds on the Ontology and Data-Driven Expert System (ODD-ES) and suggests that call-takers could benefit from applying multiple AI methods to address different inference issues and provide comprehensive analytical support. The paper analyzes emergency call handling and proposes exemplary integrations of AI methods as well as a mechanism for calculating the reliability of inferences based on experiences in similar situations.

Another study (Tollinton et al., 2020) investigates the predictive power of free text notes made by call handlers in combination with Medical Priority Dispatch System (MPDS) codes. By utilizing a bag-of-words approach and vectorized text representations, the research demonstrates that incorporating unstructured free text data significantly enhances the prediction of patient conveyance compared to using structured data alone. Various ML models, including ensemble decision tree classifiers like gradient boosting machines (GBM) and random forest (RF), were employed to handle the high-dimensional data and class imbalances, giving maximum areas under the curve (AUC) of 0.63 in testing, indicating they had little or no ability to discriminate conveyance. The findings of this research work are free text notes that can inform policy decisions on augmenting clinical decision support systems (CDSS) to optimize EMS utilization.

In another research work performed by Young et al. (2016), the authors propose an automated framework for emergency response that integrates Health Evaluation Logging (HEL) and Personal Emergency Response Systems (PERS). Their work emphasizes the incorporation of fall detection mechanisms for elderly individuals, combined with a Spoken Dialogue System (SDS) within smart home environments to facilitate real-time contact with emergency services. This initiative contributes to the broader goal of enabling aging-in-place through assistive technologies, addressing key barriers such as low user adoption and the reluctance of older adults to engage with conventional push-button alert systems. The study underscores the critical role of enhancing SDS functionality to improve the timeliness and reliability of emergency interventions, thereby advancing the development of more effective and user-centric PERS solutions.

More applications were published and deployed to enhance emergency services; for example, Corti is another real-time software using AI technology and ML to analyze ongoing conversations and enable it to detect undetectable patterns in humans to identify medical situations faster (Corti, 2016). It works by alerting medical professionals to handle urgent situations. Providing appropriate instructions to take the medical emergency case is based on the software's diagnosis that comes after the caller answers the dispatcher's questions. Some of Corti's features are Real-time Audio Analysis in which the AI listens to emergency calls and detects patterns that indicate specific medical emergencies; Decision Support by providing real-time suggestions and alerts to dispatchers based on the analysis; Continuous Learning that allows the system to improve its accuracy over time by learning from past call data and outcomes.

Another application is RapidSOS Portal, a web-based tool used by the 9-1-1 emergency center that connects users' devices by such centers (RapidSOS, 2012). Users' information will be in the emergency centers' hands in any accident and obtained directly from IoT-connected devices and applications with which RapidSOS is integrated. Besides, the RapidSOS portal clusters the emergency calls from all IoT devices and applications into one map that will give an accurate location to the dispatcher. Some features of RapidSOS are Real-time Data Transmission, which sends data such as location, medical history, and crash impact information to 9-1-1 centers; Enhanced Location Accuracy, which uses advanced location technology to pinpoint the caller's exact location; Data Integration, which aggregates data from various sources, including connected devices and apps, to provide a comprehensive overview.

Another example is AlertGo, which organizations use for crisis communication (AlertGO, 2017). This app is based on a trained team to help and assist you in providing incident data. The major feature of this app is to provide quick access to the trained team for getting information, sending alert notifications, and conference calls. This app also has an interactive live location sharing feature that helps customers to share accurate locations where incident happens. This app also has default emergency plans to guide and train customers for most occurring incidents. AlertGo features Real-time Incident Monitoring, which provides live tracking and updates of ongoing incidents; Automated Resource Allocation, which uses algorithms to determine the best resources to deploy based on the nature and location of the emergency; Integration with Other Systems, it can be integrated with other emergency management systems for comprehensive data sharing and coordination.

Another example we would like to mention is the Rava 911 Suite, which uses Smart 911 Safety profiles, including the users' information (Rave Mobile Safety, 2004). When the caller has already 9-1-1 profiles visible then it is easy for them to select the profile according to his/her situation of incident and will be able to get rapid health with less response time, which ultimately results in life-saving. This app also has a chat feature in which caller and responders can communicate in real-time. This chat feature helps people with hearing and listening disorders to write their incident queries. This app also contains video call feature to share live streaming of incident's place. The features of Rave 911 are as follows: Rave Panic Button is a mobile app that lets users quickly alert 9-1-1 and on-site personnel of an emergency, providing precise location details and additional context; the Emergency Management Platform: Centralizes communication and coordination among first responders and emergency management teams.

Table 1 illustrates the proposed ECA and other comparable systems or applications, highlighting specific features outlined in the table. In line with these applications, the proposed system ECA is more than a system to be used by some users; it is much more complicated, in which it contains ML and NLP (Chowdhury, 2003) to interpret the caller's speech-to-text, then analyzes that text and predicts the best response. ECA includes some of the standard functionalities in the previously mentioned applications and overcomes some of their shortcomings. For instance, RapidSOS relies on features and services provided by existing applications and IoT devices, which might cause duplicate requests and process the same request more than once. Also, Rave 911 does not make smart predictions using the caller's voice, and there are no instructions to handle the situation, a feature supported by ECA.

**Table 1:** Comparison between ECA and other similar applications.

**Features**	**ECA**	**Corti**	**RapidSOS**	**AlertGo**	**Rave911**
Combines many departments	Yes	No	Yes	Yes	Yes
Provide instructions to the caller-handler	Yes	Yes	No	Yes	No
Smart prediction of the emergency type	Yes	Yes	No	No	No
Suggest questions to the call-handler	Yes	Yes	No	No	No
Auto-generated report	Yes	No	No	No	No
Prioritize the emergency case	Yes	Yes	Yes	No	No
Direct each emergency situation	Yes	Yes	No	No	No

## 3 Materials and methods

Our design goal is to present a highly effective solution that addresses the challenges faced by emergency call centers. These centers play a critical role in society by enhancing public safety. However, the pressure on 9-1-1 call handlers, especially when dealing with life-threatening situations, is immense. The responsibilities of a 9-1-1 call handler are complex and stressful, from the initial call receipt to the arrival of help, potentially impacting their overall performance. When calls are redirected to the appropriate department, callers often have to repeat their information, and they may also need instructions on managing the situation before help arrives to mitigate adverse effects. The vast array of situations that call handlers encounter requires extensive knowledge across various fields, which is challenging for a single individual to manage. These factors contribute to delays in emergency response times. To deal with these challenges, this research proposes Emergency Call Assistant (ECA) to support call handlers in the efficient processing of calls. The proposed smart emergency service system provides comprehensive support to call handlers to improve overall emergency response. The proposed solution comprises the following entities: Caller, Call Handler, ECA System, and Responsible Department.

*Caller:* The caller is the person who makes the emergency call. They provide essential information about the emergency and follow the instructions given by the call handler within the ECA system.

*Call handler:* the call handler is a vital part of the ECA system, responsible for receiving emergency calls, assessing situations, and making informed decisions. They use the ECA system to provide accurate recommendations and instructions to callers.

*ECA system:* ECA serves as the system's core, featuring both front-end and back-end architectures. The front end is the interface primarily accessed by the call handler. Meanwhile, the back end hosts a machine-learning model that evaluates the input provided by the call handler, delivering predictions and recommendations for managing the emergency based on the inputted and stored information. Additionally, ECA archives essential data pertinent to emergency calls, facilitating efficient retrieval and future analysis and reporting.

*Responsible department:* within the ECA system, the responsible department plays a pivotal role. It is tasked with the duty of reporting and verifying the results of emergency calls. This department is charged with evaluating the details provided by the Call Handler and executing required measures as needed. They ensure prompt implementation of any follow-up actions necessary to protect the welfare and safety of everyone involved in the emergency scenario.

**Figure 1:**
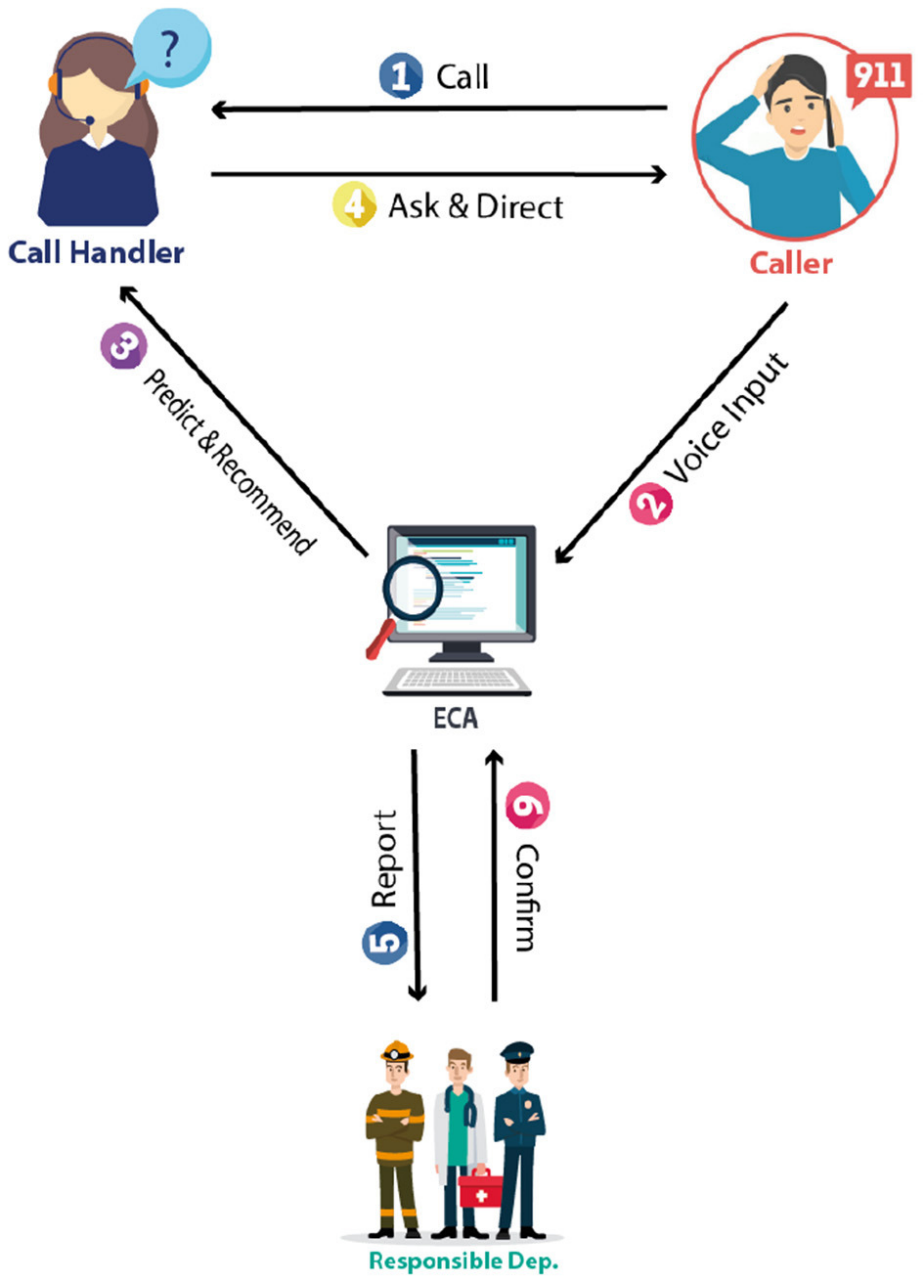
ECA system architecture.

Figure 1 shows the high-level architecture of the system, which demonstrates how the ECA functions in facilitating the handling of emergency calls and interacts with the other entities. The proposed ECA system will first process the caller's voice and convert it into a textual representation to be analyzed to extract the necessary keywords that help identify the patient's condition, predict the emergency, and prioritize the call. ECA provides life-saving instructions to the callers. Call-handler overhead is reduced because they only need to approve or suggest alternative instructions to callers. After this step, the auto-generated report will be shared with the concerned department, which ultimately results in lesser response time and provides help to the concerned department with appropriate data to act upon.

The following sub-sections will explain thoroughly the structure and design of ECA and discuss the methodology adopted for this research study to achieve the system's objectives.

### 3.1 ECA back-end design

**Figure 2:**
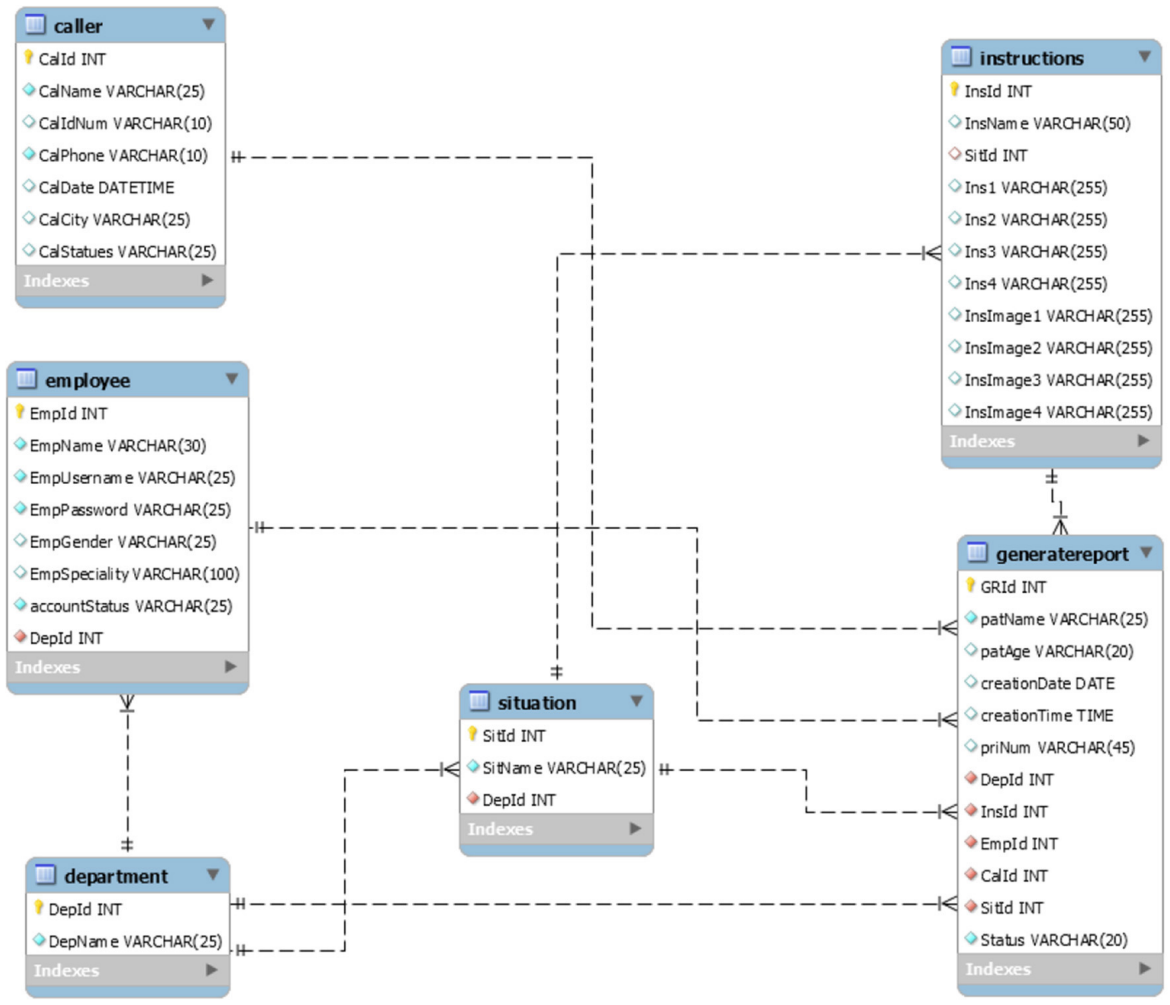
Database schema diagram.

This research makes usage of Python programming language (Python Software Foundation, 2024) to build the back end of ECA, which comprises three key components: dataset, prediction model, and database. The dataset contains relevant information and sample data for training and refining the prediction model. The prediction model utilizes ML algorithms to analyze the input from the call handler and generate predictions and recommendations for handling the emergency. The database stores crucial data related to emergency calls, enabling efficient retrieval and reference for future analysis and reporting as shown in Figure 2 of database schema.

#### 3.1.1 Dataset

Two different types of data are needed to build the ECA system: user data, which is used to build the system's basic requirements and workflow, and model data, which is used to build the ML model for training and testing to be used within the full ECA system.

**Figure 3:**
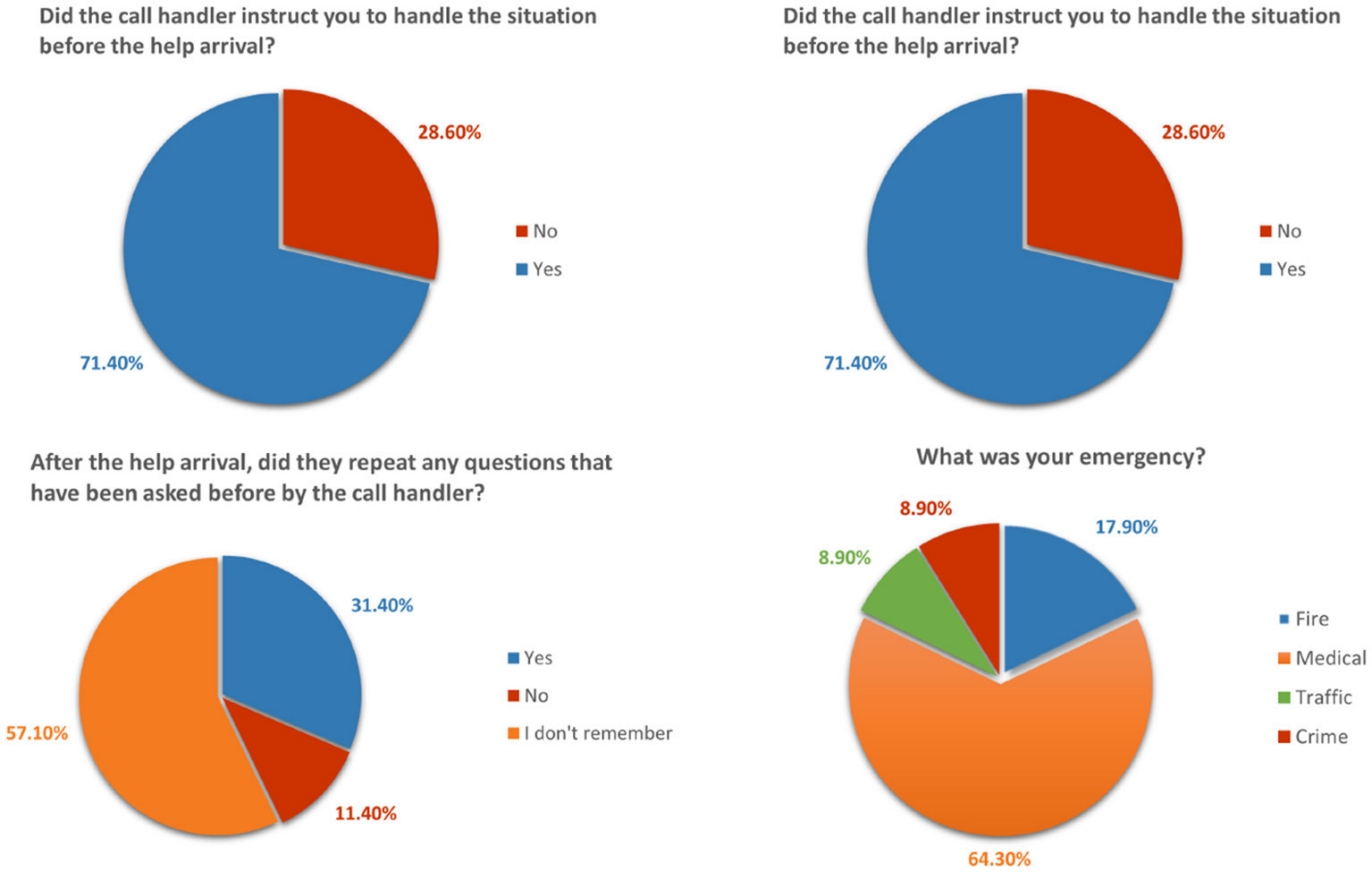
Survey questions examples with responses results.

For the user data, we surveyed to analyze the user experience and determine the need for the system and the user's requirements. Figure 3 shows examples of the questions from the survey, and here are our findings from analyzing the results:

- For proper functioning and action, the personal information of both (caller and the call handlers of 9-1-1) is essential.
- ECA software provides/suggests instructions to call handlers to get more accurate information from the callers. These suggestions play a vital role because in a high-pressure environment, no matter how well you are trained, you will miss some important points that are needed for proper action by the concerned department.
- The auto-generated report feature helps and makes correct information transfer to the concerned department easier.
- ECA system will help to facilitate handling medical emergencies since the currently used emergency software is limited to firefighting and police.

**Figure 4:**
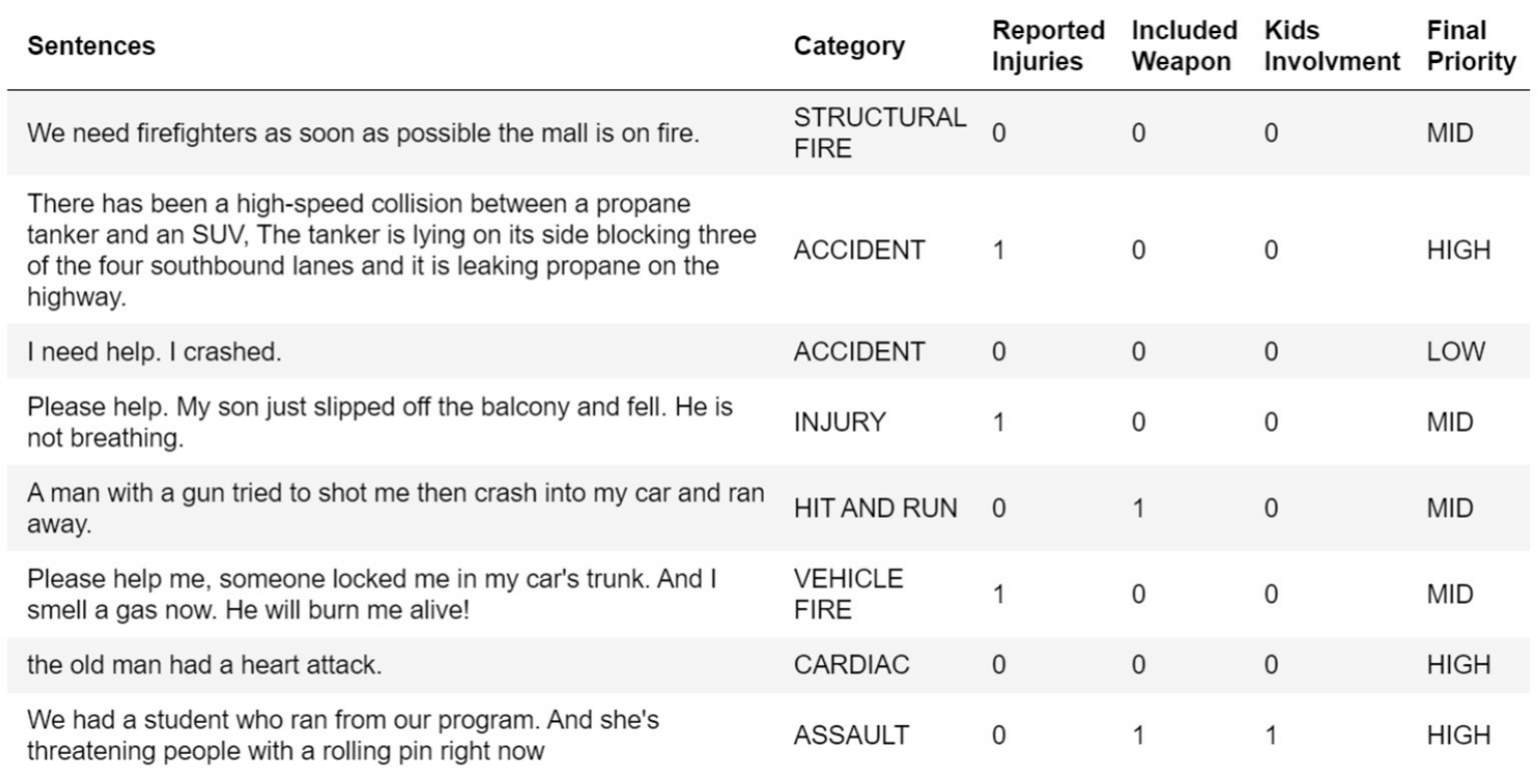
Sample of dataset.

**Figure 5:**
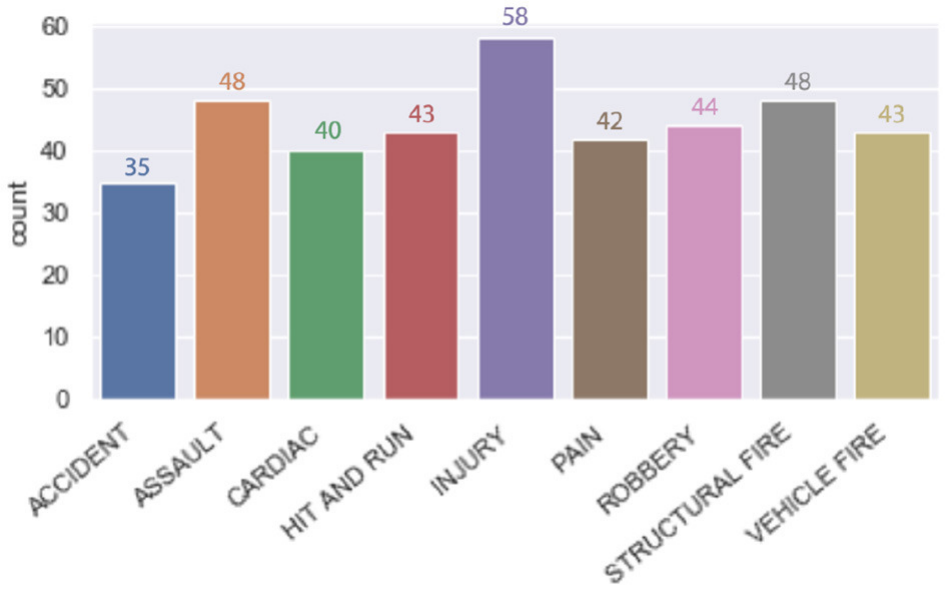
Emergencies categories.

Regarding the model dataset, we had to create a custom dataset that suited the system purpose, as we could not find a real dataset due to security reasons. We built a dataset using some useful information collected from several 9-1-1 emergency datasets, which helped us determine the emergency cases and their priority. The dataset, which is 400 samples, contains the emergency sentences that will be processed to find the emergency category/class. For example, reported injuries, reported weapons and kids' involvement are used to determine the priority, as shown in Figure 4. The number of samples in each category is equal, so the dataset is balanced to avoid any misleading performance, as shown in Figure 5. The dataset is divided into 70% training and 30% testing data.

Since that dataset is textual, we had to apply some pre-processing techniques, which involve cleaning, transforming, and preparing the data for analysis (Jurafsky and Martin, 2009). Basic text cleaning involved: lower-casing the text, punctuation removal, stop words removal, and elimination of extra spaces. Then, we had to transform and represent the text into a format that the ML model could process. Text processing involved:

- Tokenization: breaking down text into individual words.
- Part-of-speech tagging: tag each word with its appropriate part of speech, such as noun, verb and adjective.
- Lemmatization: is the task of determining that two words have the same root, despite their surface differences.
- Retokenization: joining words again into a sentence.

After processing, we represented the text into numerical format, We used Term Frequency Inverse Document Frequency (TF-IDF), which is a measure of the importance of a word to a document in a corpus, adjusted for the fact that some words appear more frequently in general. TF-IDF is used to convert the sentences into vectors based on the occurrence of words without considering the order (Rajaraman and Ullman, 2011). After the NLP, the model dataset is ready to be used by the ML model for prediction.

#### 3.1.2 ECA model

The pre-processed dataset is used to input the ML algorithm, Support Vector Machines (SVM) (Hearst et al., 1998), to predict the emergency category. SVM is a powerful ML algorithm commonly used for classification and regression tasks. The primary objective of SVM is to find an optimal hyperplane that effectively separates the data points belonging to different classes in a high-dimensional feature space. For the ECA-specific model, we utilized SVM with a linear kernel to classify the type of emergency. The linear kernel assumes that the data can be separated by a straight line, simplifying the decision boundary. Additionally, we determined the degree of closeness for the test point to be 3, allowing for a specific margin around the decision boundary. The dataset is divided into a 70–30 ratio for training and testing subsets.

Using the pre-processed dataset and the SVM algorithm, we were able to accurately predict the category of the emergency. This helps the system improve the way of handling emergency calls and provides valuable insights for quick and appropriate responses. By analyzing the data and leveraging the trained model's capabilities, the call handler will better understand the emergency, allowing the responsible department to take the necessary actions promptly.

#### 3.1.3 Database

In the ECA system, we built a relational database model with six relations shown in Figure 2. The database is used to record users, callers, departments' information, and the auto-generated reports that is produced at the end of the emergency call, and they contain applicable instructions.

### 3.2 ECA front-end design

An easy-to-use and friendly interface makes the ECA system functional and applicable to the call handlers. Electron, a framework for building desktop applications using JavaScript, HTML, and CSS, was used to develop the front end of ECA (Electron, 2024).

**Figure 6:**
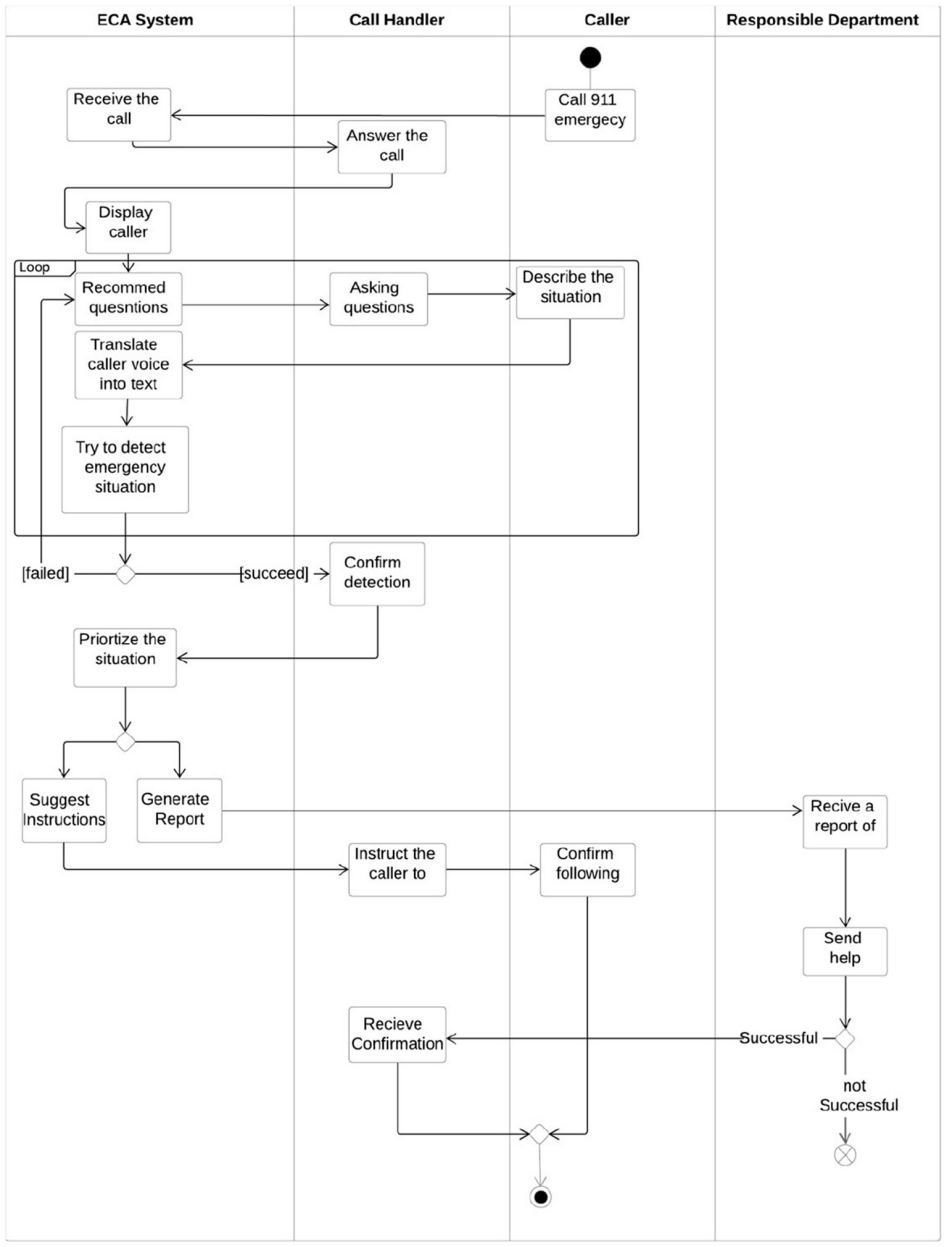
Systems activity diagram.

Figure 6 explains the flow of using the system: First, the call handler will receive the call, then the software will recommend questions according to the description of the situation, and at the same time, the conversation will be converted into text using Google Cloud Speech-to-Text API (Google Cloud, 2024). After that, some information will be extracted from the text, like the caller information, and the text will also go under multiple NLP steps to be ready to enter the trained model and predict the type of emergency. When the call handler confirms, the priority will be determined; the instructions will be shown if available to help the call handler guide the caller, and a report will be generated and sent to the responsible department to send a team to the caller.

**Figure 7:**
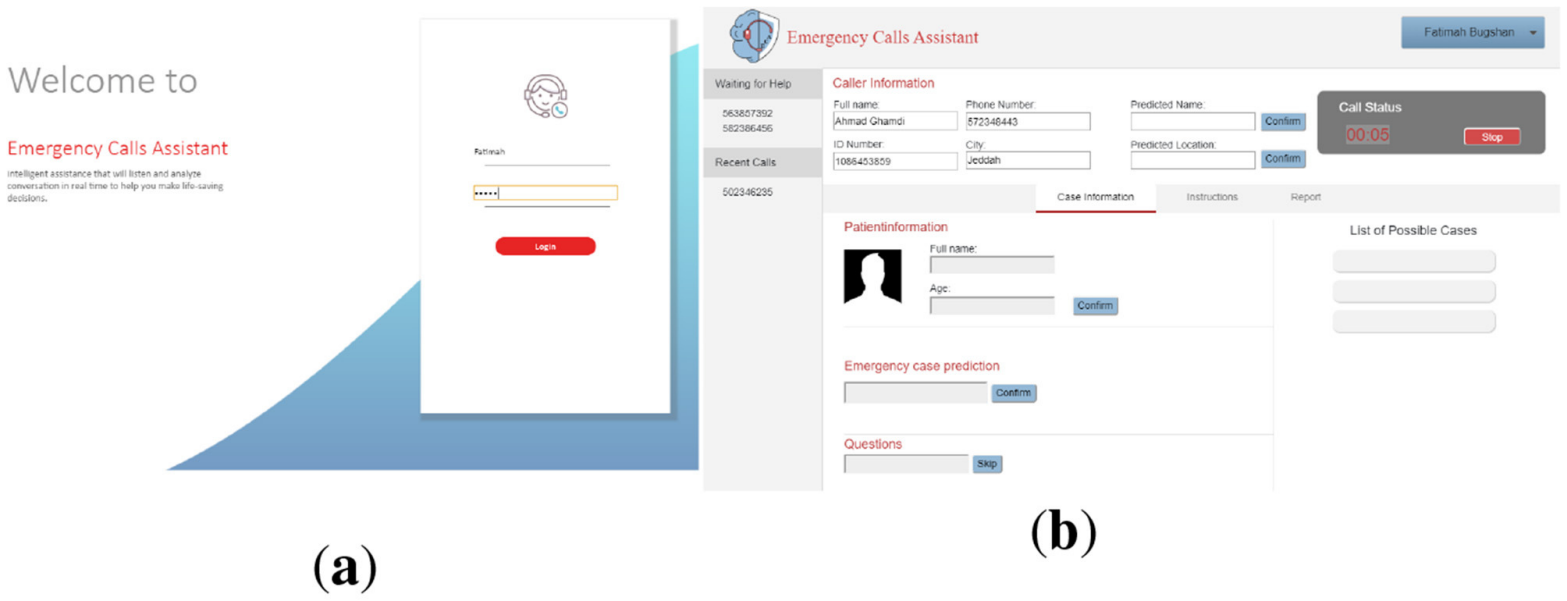
**(a)** Login interface, and **(b)** Caller information extraction.

**Figure 8:**
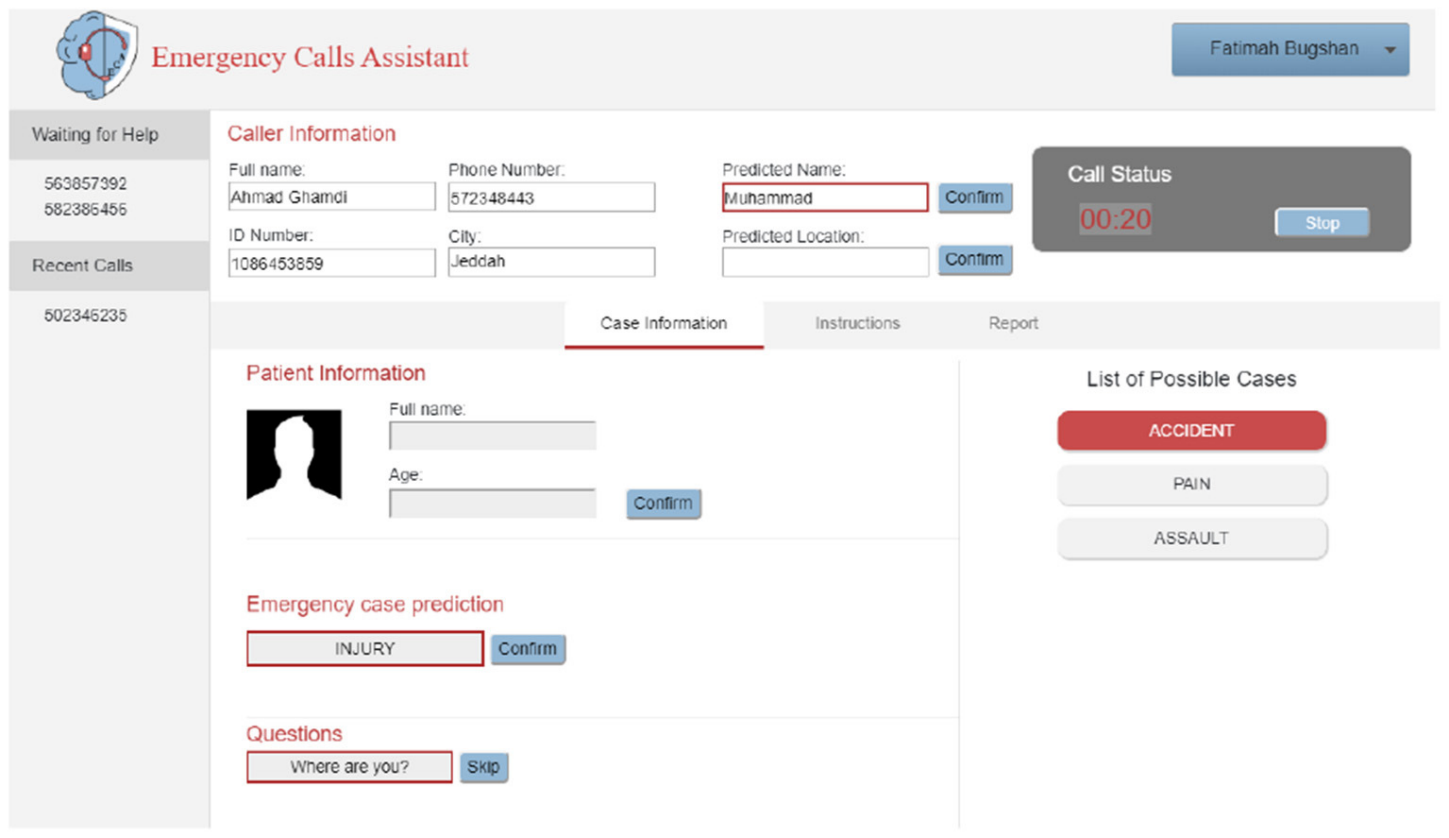
Prediction results.

The ECA system starts with the log-in interface for the call handler, as shown in Figure 7a. After recording the call, the first active caller from the waiting list is answered. When the caller describes the situation, the conversation is converted into text, and then two processes start. The first one is information extraction, such as the name of the caller and her/his or her location. That information is displayed in the caller information section on the screen, as shown in Figure 7b. The second process is emergency case prediction. The system predicted the emergency type and the result shown on the screen as illustrated in Figure 8. If the call handler does not agree with this prediction, then she/he can choose from the other possible cases on the right side of the screen (i.e., list of possible cases) as shown in Figure 8. The list is ordered based on the system classification. According to the prediction result, a question will appear to the call handler, and he/she can either use it to ask the caller or skip the question to go to the next one.

**Figure 9:**
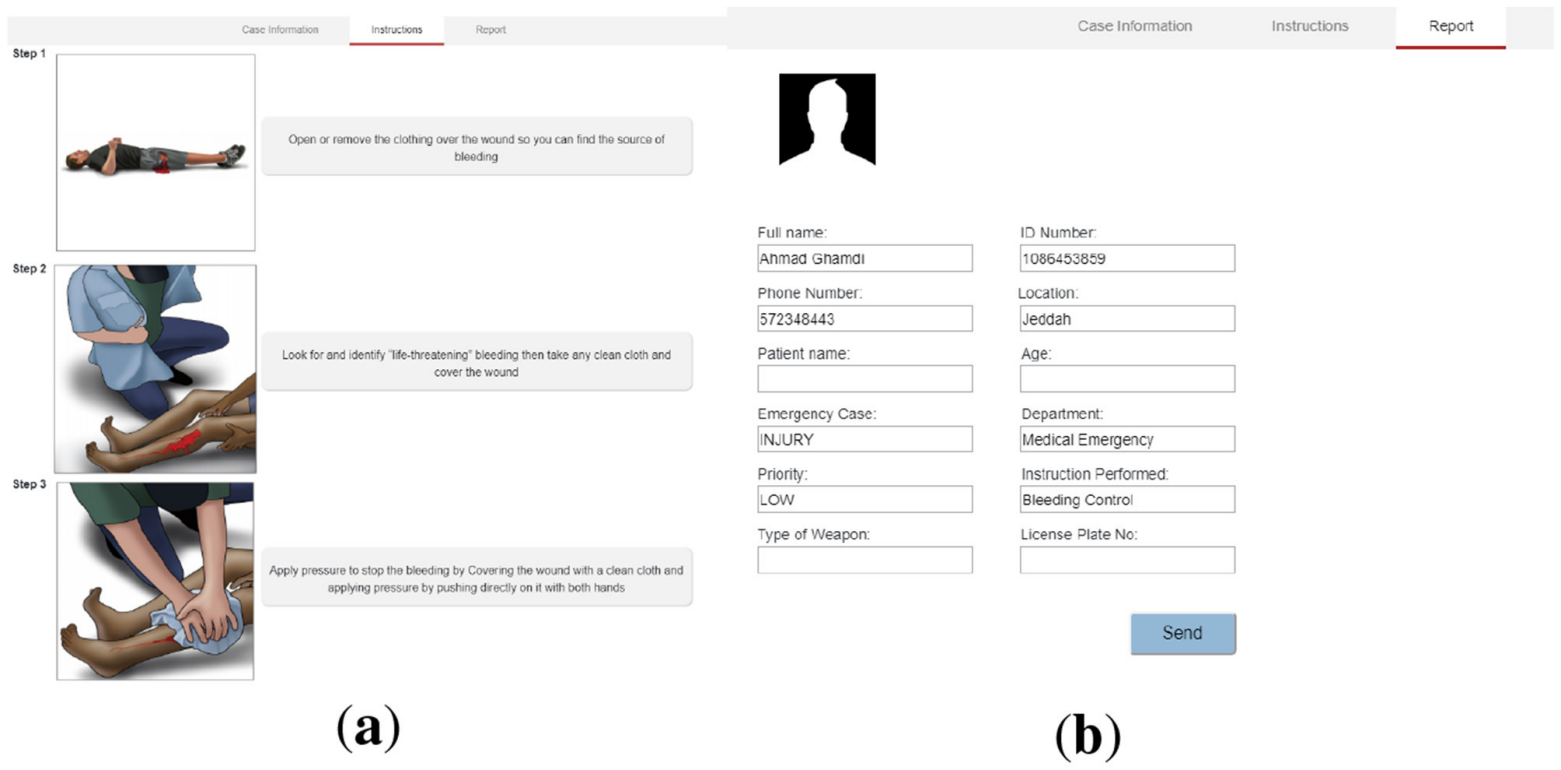
**(a)** Instructions steps and **(b)** Auto-generated report.

Moreover, the ECA system suggested steps of instructions based on the case selection to guide the caller to handle the situation before the help's arrival, as illustrated in Figure 9a. The ECA system's last step is generating a report so the call handler can send it to the responsible department, as shown in Figure 9b.

## 4 Results and discussion

To evaluate the ECA system from multiple perspectives, we test the prediction results generated by our ML model. Additionally, two techniques were utilized: unit testing and usability testing. The proposed system has been tested with many participants with a good technical background. During the testing process, we evaluated ECA functionalities and made sure that the system runs as expected.

### 4.1 First

**Figure 10:**
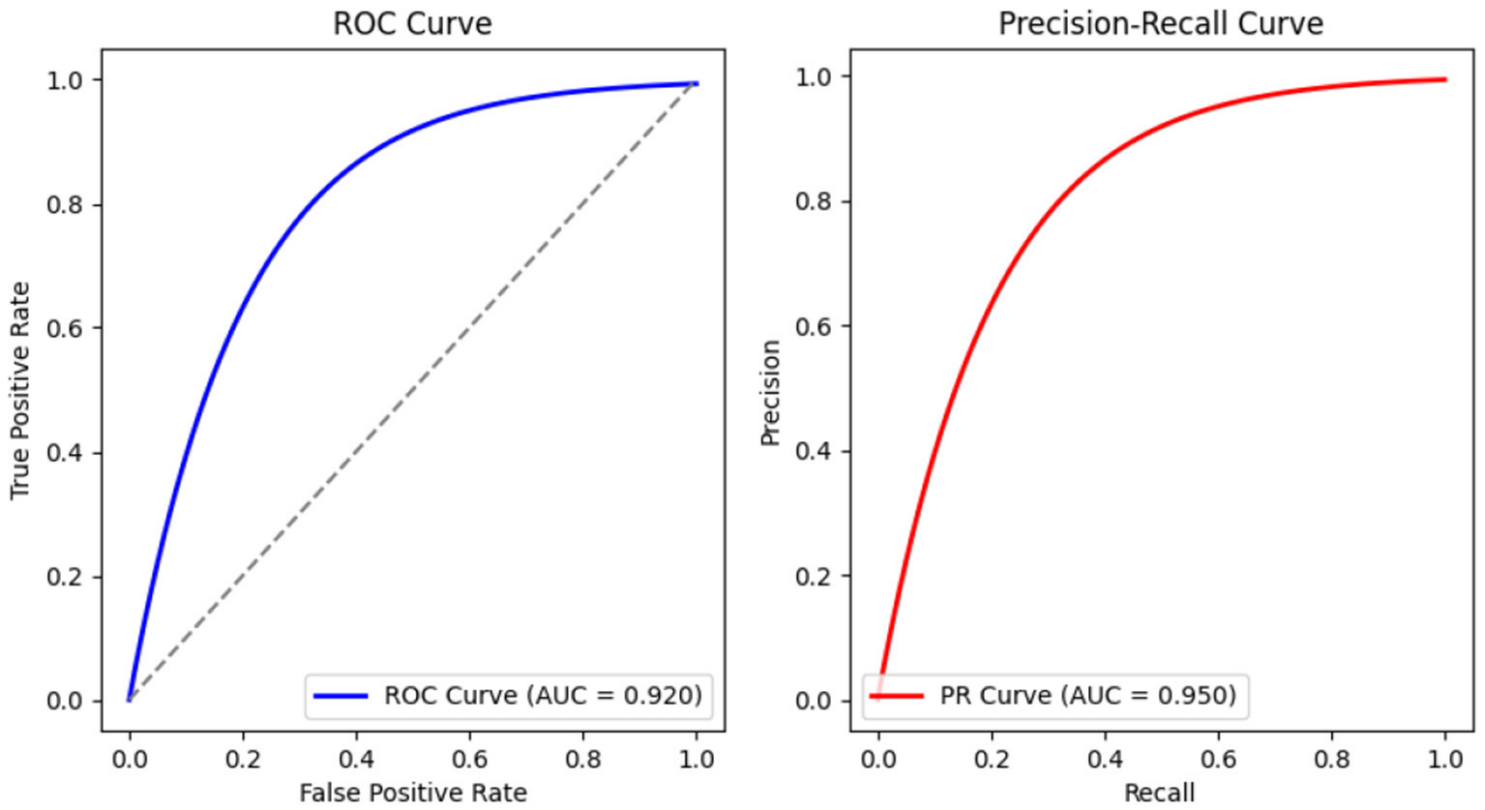
Graphs to show ROC-AUC and precision-recall curves for smooth working of proposed model.

We conducted testing on the prediction results of our implemented ML model, specifically Support Vector Machine (SVM), which was utilized to classify emergency calls into relevant categories. The classification results are 92.70% accuracy, 95.47% precision, 96.71% recall, and 96.18% F1 score, which can be calculated according to Equations 1–3. The graphs in Figure 10 show ROC-AUC and precision-recall curves for the smooth working of the proposed model.


(1) $$\begin{array}{l} Accuracy=\frac{Tp+Tn}{Tp+Tn+Fp+Fn} \end{array}$$

(2) $$\begin{array}{l} Precision=\frac{Tp}{Tp+Fp} \end{array}$$

(3) $$\begin{array}{l} Recall=\frac{Tp}{Tp+Fn} \end{array}$$

where *Tp, Tn, Fp*, and *Fn* denote the number of true positive, true negatives, false positives, and false negatives, respectively.

The accuracy of the ECA model can be attributed to several factors inherent to the study's current state. However, the significance of our findings and contributions remains impactful. Firstly, the dataset used for training and testing was custom-built due to the unavailability of real-world data for security reasons. The dataset is balanced and pre-processed and represents less diverse scenarios that can be faced in actual emergency calls. Secondly, the SVM model employed with a linear kernel may not capture the complexities and non-linear relationships within emergency call data. Furthermore, to build the foundation, this research incorporates hand-crafted features like TF-IDF that do not capture semantic information. The integration of state-of-the-art word embedding techniques like BERT, GLOVE, and FastText could improve the results. Despite these limitations, the proposed approach highlights the critical need for automated systems in emergency services and provides a strong starting point for other researchers to work on this domain. Considering the complex nature of emergencies that require continuous adaptation and learning, this work lays the groundwork for such advancements.

### 4.2 Second

The performance of each component in the development of this framework is judged using a unit testing framework. This test also confirms that each unit is functioning properly and playing its part in framework designing and assisting call handlers. This also identifies and resolves any potential problems that may arise during the testing process (Yu et al., 2021).

### 4.3 Third

The usability of the ECA system is tested by giving the users some tasks to perform. The following three main criteria have been used to determine the usability of the system, according to Jakob Nielsn (Nielsen, 2012).

- Learnability: it refers to the ease with which users can understand and navigate the solution to accomplish basic tasks.
- Efficiency: it refers to how quickly they can perform tasks once users have become familiar with the design.
- Satisfaction: it reflects the user's response to the design and its pleasantness during use.

Two test methods were employed in this part. Participants were asked to interact with the proposed system interfaces and complete a survey for each task, which assessed the criteria mentioned above. The system's usability was evaluated through six main tasks, and the results are presented in Table 2, indicating whether each objective was achieved or not.

**Table 2:** Tasks achievement.

**Number**	**Name**	**Achieved/not achieved**
Task 1	Predict the emergency case type	Achieved
Task 2	Suggest questions	Achieved
Task 3	Provide instructions	Achieved
Task 4	Predict all the responsible departments	Not Achieved
Task 5	Auto-generate report	Achieved

Upon analyzing the recorded responses for the system usability questions, the average results indicate that 80% of the responses achieved an excellent level of task accomplishment in terms of learnability. Additionally, 20% of the responses demonstrated an acceptable level of achievement. Regarding efficiency, 70% of the responses achieved an excellent level of task accomplishment, while 20% demonstrated an acceptable level of achievement. In terms of satisfaction, 90% of the responses achieved an excellent level of task accomplishment, while 10% demonstrated an acceptable level of achievement.

### 4.4 Comparison with SOTA approaches

To validate the performance of the proposed model with other SOTA learning models, we tested several other learning models on the same approach. We have tested GBC, RF, LSTM, and CNN models with the proposed SVM. The details of the classifiers are shared in the subsection below and results are shared in Table 3.

**Table 3:** Comparison of proposed framework with SOTA models.

**Model**	**Accuracy**	**Precision**	**Recall**	**F1 score**
RF (word embedding)	88.57	87.67	88.04	87.71
RF (TF-IDF)	90.81	92.42	92.94	92.72
GBC (word embedding)	91.11	90.43	90.95	90.73
GBC (TF-IDF)	89.83	89.34	89.55	89.45
LSTM (word embedding)	86.52	87.35	87.44	87.39
CNN (word embedding)	91.31	91.38	91.78	91.58
Proposed SVM (word embedding)	91.51	92.38	93.52	93.08
Proposed SVM (TF-IDF)	92.70	95.47	96.71	96.18

#### 4.4.1 Gradient boosting classifier

GBC is an ensemble learning model that combines multiple weak learners to collectively minimize the loss function during the training process (Umer et al., 2022). GBC follows an iterative approach to build an additive model, where current weak learners remain unchanged with the addition of a weak learner at each repetition. GBC uses DTs as weak learners, and the prototypical GBC is built by adding DTs iteratively.

#### 4.4.2 Random forest

RF is an ensemble model rooted in tree-based methodology, serving both classification and regression tasks. The process involves fitting multiple DTs during the learning phase and subsequently consolidating their forecasts through majority voting (Breiman, 1996). The final predicted class is determined by the consensus of a higher number of DTs. To construct these trees, RF employs algorithms like the Gini index and Entropy, which identify significant features to form the tree structure.

#### 4.4.3 Long short-term memory (LSTM)

This model is a type of RNN model that is particularly effective in catching temporal dependencies in sequential data (Cascone et al., 2023). LSTMs address the limitations of traditional RNNs by incorporating a cell memory block that can maintain information of long sequences and prevent issues like vanishing and exploding gradients. This makes LSTMs highly suitable for tasks such as time series prediction, natural language processing, and network attack detection, where understanding the context from previous inputs significantly enhances performance.

#### 4.4.4 Convolutional neural network

A CNN's convolutional layers and pooling layers make it particularly suitable for capturing complex features (Yamashita et al., 2018). The strength of CNNs lies in their ability to undergo end-to-end training, which simultaneously optimizes all network parameters. This comprehensive training approach enhances the network's robustness against variations in input and noise, ensuring reliable predictions. CNNs function as feed-forward networks where convolutional layers apply filters to the outputs of preceding layers, enhancing data processing. These networks are structured with several key components: activation layers, pooling layers, dropout layers, and fully connected layers. Pooling layers play a crucial role by simplifying and refining the features, reducing their spatial dimensions through strategies like average or maximum pooling. The fully connected layers are essential for making the final decisions in the network. Dropout layers help mitigate the risk of overfitting by randomly deactivating certain neurons during training. Activation functions within these layers are critical for determining the significance of the processed inputs.

#### 4.4.5 Discussion analysis

From Table 3, it can be observed that the proposed SVM model outperforms all other traditional machine and deep learning models in terms of accuracy, precision, recall, and F1 score with results of 92.70%, 95.47%, 96.71%, and 96.18%. The second-best performing model is CNN with 91.31% accuracy. To ensure robustness and reliability of the proposed SVM-based classification model, we conducted 10-fold stratified cross-validation and report a mean accuracy of 92.35% with a 95% confidence interval of (91.35%, 94.05%). In addition, we compared the SVM model against a Gradient Boosting Classifier (GBC) baseline using a paired *t*-test, yielding a *p*-value of 0.0043, indicating statistical significance. These results demonstrate not only high performance but also statistical confidence and stability of the proposed framework. To gain insight into the limitations of our model, we analyzed misclassified records and identified that accidents and hit-and-run records are most frequently misclassified due to overlapping situations. The deeper insights show that the wording similarities contribute to misclassification. Representative errors are manually reviewed. This analysis motivates future work on incorporating deeper contextual modeling and temporal cues from conversation history.

### 4.5 Real-time evaluation of proposed framework

To ensure practical deployability, this research evaluated the framework's end-to-end latency and concurrent processing capacity on a standard CPU setup (Intel i7, 16GB RAM). The average speech-to-text latency per call is 1.87s, text preprocessing and feature extraction took 0.21s, and SVM inference required 0.05s, yielding a total latency of ~2.13s per call. In concurrent testing, the system processed 10 parallel calls in 5.6 s, averaging 0.56 s per call. CPU usage peaked at 45%, and memory usage remained under 60%, indicating the system is lightweight and suitable for deployment in real-time 911 dispatch applications.

### 4.6 Significance of the proposed framework

To check the significance of the proposed framework, we have tested it on another independent dataset (Teitelbaum, 2023) named as “9-1-1 Recordings: The First 6 Seconds.” The dataset contains the first 6 seconds of call recordings of 700 people called to 9-1-1. The dataset is in .wav form. Some emergency calls are also from airplane crashes. The proposed framework gives 97.34% accuracy, 96.91% precision, 97.98% recall with 97.91% F1-score.

## 5 Conclusions and future work

The Emergency Calls Assistant (ECA) system represents a significant breakthrough in the field of emergency response services. Through the integration of cutting-edge AI and NLP technologies, the ECA system enhances the effectiveness of call handling, improves decision-making, and streamlines emergency procedures. The results of the performance evaluation demonstrate the system's potential to revolutionize emergency call handling and ensure timely and appropriate assistance to callers. Among many other benefits of the ECA system, the major one is its ability to provide pre-arrival instructions to 9-1-1 callers. Using real-time analysis of call content, ECA manages the situation until help arrives. This key feature not only helps in decision-making but also ensures the well-being and the safety of people in distress. Furthermore, the ECA system generates auto-filled incident reports, streamlining the process and reducing response times. These reports are shared with relevant departments, removing the overhead of manual report creation and ensuring that crucial information that was conveyed by the person on call is listed properly in the report. The high-pressure nature of emergency call handling can lead to errors. However, the ECA system significantly reduces the likelihood of errors by providing call handlers with real-time analysis and classification of call content. This feature assists call handlers in navigating diverse emergency scenarios where extensive knowledge across various fields is often required. As future work, ECA can be improved in many ways to have more efficient and faster services, like utilizing a larger and real dataset, enhancing the classification results, adding more emergency cases, Adding location detection of the caller. Furthermore, in the model development section, we can improve the model performance using state-of-the-art word embedding techniques with a transformer architecture. The multimodal input for scene understanding can also be the framework to much extent.

## Data Availability

The original contributions presented in the study are included in the article/Supplementary material, further inquiries can be directed to the corresponding author.
