# Supplementary material for: AI-powered smart emergency services support for 9-1-1 call handlers using textual features and SVM model for digital health optimization
Source: Front Big Data. 2025 Jul 7;8:1594062. doi: 10.3389/fdata.2025.1594062 (PMC12277377; doi:10.3389/fdata.2025.1594062)
Supplement: Supplementary file 1 [file Table_1.doc]

import speech_recognition as sr

import pandas as pd

import re

from sklearn.feature_extraction.text import CountVectorizer, TfidfVectorizer

from sklearn.svm import SVC

from sklearn.model_selection import train_test_split

from sklearn.pipeline import FeatureUnion, Pipeline

from sklearn.metrics import classification_report, accuracy_score

from sklearn.preprocessing import LabelEncoder

# Step 1: Convert Voice Call (WAV) to Text

def voice_to_text(audio_file_path):

    recognizer = sr.Recognizer()

    with sr.AudioFile(audio_file_path) as source:

        print("Listening to call...")

        audio = recognizer.record(source)

        try:

            text = recognizer.recognize_audio(audio)

            print("Transcription:", text)

            return text

        except sr.UnknownValueError:

            print("=Speech Recognition could not understand audio")

            return ""

        except sr.RequestError as e:

            print(f"Could not request results; {e}")

            return ""

# Example: call_text = voice_to_text("path_to_audio/911call.wav")

call_text = voice_to_text("911_call_example.wav")  # Replace with your actual .wav file

# Step 2: Preprocess the transcribed text

def preprocess(text):

    text = text.lower()

    text = re.sub(r'[^a-z\s]', '', text)

    return text.strip()

processed_call_text = preprocess(call_text)

# Step 3: Sample dataset (replace with real transcribed call records)

data = {

    'call_transcript': [

        "There's a fire in my building! Smoke everywhere!",

        "I just witnessed a car accident on Main Street.",

        "My father is not breathing, we need an ambulance!",

        "Someone broke into my house and stole everything.",

        "My neighbor is screaming for help, it might be domestic violence.",

        "There’s a person passed out in the park!",

        "A man is holding a knife threatening people downtown."

    ],

    'emergency_type': [

        'Fire', 'Accident', 'Medical', 'Burglary', 'Violence', 'Medical', 'Violence'

    ]

}

df = pd.DataFrame(data)

df['processed_text'] = df['call_transcript'].apply(preprocess)

# Step 4: Encode labels

le = LabelEncoder()

df['label'] = le.fit_transform(df['emergency_type'])

# Step 5: Split and prepare data

X_train, X_test, y_train, y_test = train_test_split(df['processed_text'], df['label'], test_size=0.3, random_state=42)

# Step 6: Feature Engineering

tf = CountVectorizer(ngram_range=(1,2), stop_words='english')

tfidf = TfidfVectorizer(ngram_range=(1,2), stop_words='english')

combined_features = FeatureUnion([

    ("tf", tf),

    ("tfidf", tfidf)

])

# Step 7: Build and Train Model

pipeline = Pipeline([

    ('features', combined_features),

    ('classifier', SVC(kernel='linear', probability=True))

])

pipeline.fit(X_train, y_train)

# Step 8: Classify New Transcribed Call

if processed_call_text:

    predicted_label = pipeline.predict([processed_call_text])[0]

    predicted_class = le.inverse_transform([predicted_label])[0]

    print(f"Predicted Emergency Type: {predicted_class}")

# Step 9: Optional - Evaluate on test set

y_pred = pipeline.predict(X_test)

print("\nModel Evaluation:\n", classification_report(y_test, y_pred, target_names=le.classes_))
